# Annotations

**Published:** 1901-08-17

**Authors:** 


### ANNOTATIONS.

### The Registration of Midwives.

It was only natural where so many medical practitioners were gathered together as there were lately at Cheltenham, that the question of the midwives should attract a considerable amount of attention. At a meeting which was addressed by the direct representatives on the General Medical Council and others, various views were maintained. Dr. Glover had, however, a great advantage in that he knew what he wanted. He held that some regulation of the practice of midwives was urgently required, that the practice of unlicensed midwifery should be stopped and penalised, that the midwife should be required on the occurrence of any irregularity or abnormality of mother or child to apply to a registered medical practitioner, that the local sanitary authority of each district should be required to pay to medical men a reasonable fee (not less than a guinea) when so appealed to by the midwife. Surely this is a reasonable programme. To show how divided the profession is, however, on this subject, and how hopeless it is to expect anything like unanimous action in the matter we may refer to a letter from Mr. G. H. Broadbent, of Manchester, who says that what the opponents of the Midwives' Registration Bill want is "for the medical acts to be carried out, that no one should be allowed to practice midwifery upon their own responsibility, except those who are qualified and registered in that subject as well as in medicine and surgery," while Mr. George Brown, at the meeting above referred to, said that he opposed the present Bill *in toto*, and was in favour of a Bill for the better training of nurses for poor people which he believed would "meet the case." Whenever the matter shall come seriously before Parliament we may be pretty certain that it will be decided with but little deference to a profession so divided within itself.

### The Colour Cure.

The author of a little book that has come into our hands, starting on the fact that one rarely sees a dead donkey and that in regard to men we have the spectacle of people filled with disease and of a land crowded with hospitals, comes to the conclusion that "too many drugs are taken." Yet, according to our author there is a way, even without nauseous drugs, if men would but take it. For is there not chromopathy? It is well known, we are told, that in nature heat associates itself with red. Fire is of this colour; so, too, are capsicum, cloves, musk, balsam of Peru, and other plants used as drugs, the tints varying from bright red to dark brown. Again, in diseases heat is expressed by redness, as in inflammations, "and as regards the emotions, people turn red with passion." Blue, on the other hand, is usually associated with cold. Do not people go blue with cold? Evidently red is heating and blue is cooling. The therapeutic utility of this wonderful discovery is obvious enough, and it is not surprising that with such a solid groundwork to go upon the chromopathists find that the two most important colours for their purpose are red and blue. Red should be used, we are told, where there is a lack of vitality in the system, whereas blue is applied in all conditions of inflammation. All this appears very simple. The other colours, however, are rather more puzzling, but we are assured that yellow is a capital cerebral stimulant, and an emetic or laxative, while combined with red and thus forming orange a powerful specific is provided "in cases of cold and dormant or chronic conditions." As for the manner of the treatment it is very easy, and can be made more simple still by the exercise of a little faith. The apparatus required consists of several sheets of coloured glass, or if one happens to know what colour is wanted of course one sheet of that colour is enough. The glass, framed or not, may be hung up in the window where the sun comes in, in such a way that one can sit in the coloured light so produced for from twenty minutes to an hour. But all this sitting in the sunlight is a bother, so the inventors of chromopathy, well knowing the inveterate fondness of sick man for something in the form of physic —something that he can swallow — have with great astuteness discovered that bottles of plain water exposed to these colours become endowed with their proper virtues. Thus it is a very simple plan to go about one's business and leave one's water bottle in the window exposed to the required colour, and then a couple of tablespoonfuls twice a day will do the trick. A bath in colour water is also said to be useful. One's clothes again may be made to have a therapeutic effect by being hung in coloured light, while our food may be treated in the same way, which is said to be "a pleasant way of taking medicine." And all this rubbish is a therapeutic system which is solemnly proposed in all seriousness as a means of treating disease!

### The Toothsome Sausage.

August is certainly not the month in which sausages would appeal to us as a wholesome and dainty kind of food. The very seasoning by which these delicacies are made tempting to certain palates is capable of much in the way of covering up those tastes and odours by aid of which commencing putrefaction is made obvious to the senses. A tale has lately been going round the papers which illustrates at once the baseness of human nature and the infinite capacity possessed by an ordinary sausage skin for covering up the nature of things. At the best of times, and with the most honourable of butchers, sausages are but a conglomeration of bits and scraps, mingled sometimes with joints which, to say the least of it, are approaching the borderland of freshness. But it seems to have been discovered by some astute manufacturers in the East of London that it is a waste of good sausage skins to fill them with good meat when cats' meat is available. So a little trade has grown up between the cats' meat man and the sausage maker. The sausage machine is to these good people as the melting pot is to the receiver of stolen spoons. Good meat and bad, beef, mutton, and pork, cats' meat, dogs' meat, and, perhaps even dogs themselves may go in, but all comes out alike, and, by aid of a little condiment, is made food for man. A great cry has lately arisen for a far more complete inspection of butcher's meat than is possible under present conditions of slaughter. But after all a judicious purchaser can tell something about the nature of the joints he buys. About the sausages (granting that a judicious purchaser ever does buy sausages) he can tell nothing, and it would be easy to maintain that of all things the making of which requires inspection, sausages ought to stand *facile princeps*. To condemn a whole carcase of good, sound-looking meat because there happen to be some bits of tuberculous matter here and there is an admirable proceeding, and shows how far we are willing to go in maintaining at any cost a high standard of wholesomeness in the food of the people. But it is rather hard after all this to find that our sausages are stuffed with cats' meat!

